# Reliability, validity, and responsiveness of three scales for measuring balance in patients with chronic stroke

**DOI:** 10.1186/s12883-018-1146-9

**Published:** 2018-09-13

**Authors:** Ahmad H. Alghadir, Einas S. Al-Eisa, Shahnawaz Anwer, Bibhuti Sarkar

**Affiliations:** 1 Rehabilitation Research Chair, College of Applied Medical Sciences, King Saud University, P.O.Box-10219, Riyadh, 11433 Saudi Arabia; 2 National Institute for Locomotor Disabilities (Divyangjan), Kolkata, India

**Keywords:** Dynamic gait index, Stroke, Balance, Berg balance scale, Rehabilitation, Timed up and go test

## Abstract

**Background:**

Various outcome measures are used for the assessment of balance and mobility in patients with stroke. The purpose of the present study was to examine test-retest reliability, construct validity, and responsiveness of the Timed Up and Go Test (TUG), Berg Balance Scale (BBS), and Dynamic Gait Index (DGI) for measuring balance in patients with chronic stroke.

**Methods:**

Fifty-six patients (39 male and 17 female) with chronic stroke participated in this study. A senior physical therapist assessed the test-retest reliability and validity of three scales, including the DGI, TUG, and BBS over two testing sessions. In addition, the third assessment of each scale was taken at the time of discharge to determine the responsiveness of the three outcome measures.

**Results:**

The reliability of the TUG (intraclass correlation coefficient [ICC_2,1_] = 0.98), DGI (ICC_2,1_ = 0.98) and BBS (ICC_2,1_ = 0.99) were excellent. The standard error of measurement (SEM) of the TUG, DGI, and BBS were 1.16, 0.71, and 0.98, respectively. The minimal detectable change (MDC) of the TUG, DGI, and BBS were 3.2, 1.9, and 2.7, respectively. There was a significant correlation found between the DGI and BBS (first reading [r] = 0.75; second reading [r] = 0.77), TUG and BBS (first reading [r] = −.52; second reading [r] = −.53), and the TUG and DGI (first reading [r] = 0.45; second reading [r] = 0.48), respectively.

**Conclusions:**

The test-retest reliability of the TUG, BBS, and DGI was excellent. The DGI demonstrated slightly better responsiveness than TUG and BBS. However, the small sample size of this study limits the validity of the results.

## Background

Stroke is the common cerebrovascular disease with a high mortality rate and persistent disability in adults worldwide [1]. The prevalence of stroke in Saudi Arabia is relatively low compared to the Western and Asian countries [2]. A balance disorder is the commonest cause of disability in patients with stroke [3]. Previous studies have reported an increased postural sway, asymmetrical weight distribution, reduced stance capability, and impaired weight shifting ability in individuals with stroke [4–6]. These problems can impair function and activities of daily livings [7]. Therefore, interventions for enhancing balance and functional mobility is the focus of rehabilitation for the people with chronic stroke [7]. In addition, maintaining balance has been found to be a strong predictor of independent living [8] and was highly correlated with the perceived disability at the time of discharge from the rehabilitation [9]. Assessment of the balance can assist the therapists in the diagnosis, selection of appropriate interventions, and outcome measurements [10].

Various outcome measures are used for the assessment of balance and mobility in patients with stroke [7, 11–17]. The Timed Up and Go Test (TUG), Berg Balance Scale (BBS), and Dynamic Gait Index (DGI) are reliable and valid scales that clinicians commonly used to evaluate the functional abilities of lower limbs in patients with stroke. Flansbjer et al. [18] reported that the TUG test is a single-task measure involves a single 180-degree turn and straight pathway walking. In a systematic review study, Pollock et al. [19] reported that the multiple-task measure was better than a single-task measure in evaluating balance. However, multiple-task outcome measures often take a long time and could not detect specific balance deficits [20].

Previous studies suggested that impairments in the multiple tasks balance function indicates negative outcomes for instance, increased risk of fall [21–23] and reduced physical and cognitive function [24–26]. Similarly, other studies reported reduced postural stability while performing simultaneous activities of two or more balance tasks [27, 28]. Thus, assessment of balance function while performing two or more balance tasks concurrently is critical for rehabilitation of patients with stroke.

Therefore, the present study aimed to compare the single-task outcome measure such as TUG test with the multiple-task outcome measures, including the BBS and DGI for measuring balance and mobility in patients with chronic stroke.

## Methods

### Participants

Fifty-six patients with chronic stroke from the outpatient physiotherapy department were participated in the study. The inclusion criteria were as follows: first episode of stroke (more than 3 months of duration since onset), able to follow simple instructions, absence of comorbidities (e.g. fracture, brain tumor, severe rheumatoid arthritis, or amputation), and able to walk at least 10 m (assessed by the examiner to confirm the eligibility), with or without an assistive gait device. The institutional ethics committee, Rehabilitation Research Chair, King Saud University, Riyadh, Saudi Arabia, approved this study. An informed consent form was signed by each participant.

### Procedures

A senior physical therapist administered the BBS, DGI, and TUG tests. The BBS was developed to evaluate the balance performance and determine the fall risk in the elderly [29]. The BBS measures multi-tasking ability and includes 14 items that require participants to maintain their balance in different tasks and positions with various levels of difficulty. Each item is scored from 0 to 4 points (best possible score, 56). The inter-rater and intra-rater reliability of BBS for the patients with stroke was 0.97 and 0.98, respectively [30]. There is a high risk of falling if the score is 44 or less [30].

The DGI was designed to evaluate the dynamic balance during walking [31]. It has eight items that require participants to maintain balance during normal walking and walking with different situations (e.g., changing speed, head turn, over and around the obstacles, pivot turn, and stairs climbing). Each item is scored from 0 to 3 points (best possible score, 24). A higher total DGI score signifies a higher level of independent functional mobility. The DGI was correlated with the BBS and Activities-specific Balance Confidence Scale (ABC) [29, 32].

The TUG test is designed to measure functional mobility [33]. The test-retest reliability of the TUG was excellent for individuals with stroke (ICC = 0.95) [34]. Duration of ≥13.5 s on the TUG was associated with an increased fall risk in the elderly and persons with vestibular dysfunction [35].

The BBS, DGI, and TUG tests were administered by a single rater in two testing sessions over a period of 1 week, to assess the test-retest reliability. In addition, the third assessment of each scale was taken at the time of discharge to determine the responsiveness of the three outcome measures. The duration of the entire testing procedure was 45–60 min.

### Statistics

Descriptive data, including mean and standard deviation (SD) values for each score distribution, were presented for each scale. Test-retest reliability of the TUG, total DGI scores, and BBS scores were analyzed using the ICC_2,1_. The agreement between two readings of each scale was assessed using the Bland-Altman plot method [36]. The mean of the scores on the x-axis was plotted with the difference of scores on the y-axis [36]. The standard error of measurement (SEM) was determined by the following formula: SD √(1− ICC) [37]. The minimum detectable change (MDC) was determined by the following formula: 1.96*√2*(SEM) [38]. In addition, the construct validity of the three outcome measures was assessed using the Pearson’s correlation coefficient test. Furthermore, the responsiveness of the three outcome measures to change from baseline to discharge was determined using the standardized response mean (SRM). The magnitude of responsiveness was considered as follows: an SRM > 0.8 is large, 0.5 to 0.8 is moderate, and 0.2 to 0.5 is small [39]. A *p*-value of ≤0.05 was set for the statistical level of significance. All statistical analyses were done using the statistical package for the social sciences for Windows version 22 (IBM Inc., Chicago, Illinois, USA).

## Results

Table 1 details the demographic data and stroke-related characteristics. The majority of the participants were male (70%). Right-sided hemiplegia was present in 59% of the participants. There were no significant differences in the mean TUG score, total DGI score, and BBS scores between measurements (Table 2). There was no history of other episodes of stroke during rehabilitation period in any patients.

**Table 1:** Participant’s characteristics

	*N* = 56
Sex, male/female	39/17
Age (mean ± SD), years	58.6 ± 9.8
Height (mean ± SD), cm	165.1 ± 5.8
Weight (mean ± SD), Kg.	63.7 ± 6.5
Affected side, right/left	33/23
Duration since onset (mean ± SD), Months	22.2 ± 18.3
Stroke type, infarction/hemorrhage, n	32/24

**Table 2:** Test Scores of the timed up and go test (TUG), the dynamic gait index (DGI), and the berg balance scale (BBS)

	TUG_1_	TUG_2_	DGI_1_	DGI_2_	BBS_1_	BBS_2_
Mean ± SD	20.1 ± 8.3	19.8 ± 8.1	14.9 ± 5.3	15.3 ± 5.3	41.4 ± 10.9	41.8 ± 10.9
Range (minimum - maximum)	8.0–44.5	8.0–43.8	4–24	4–26	5–56	9–56
*p-value	0.2139		0.0618		0.0874	

*Paired t-test

**Fig. 1:**
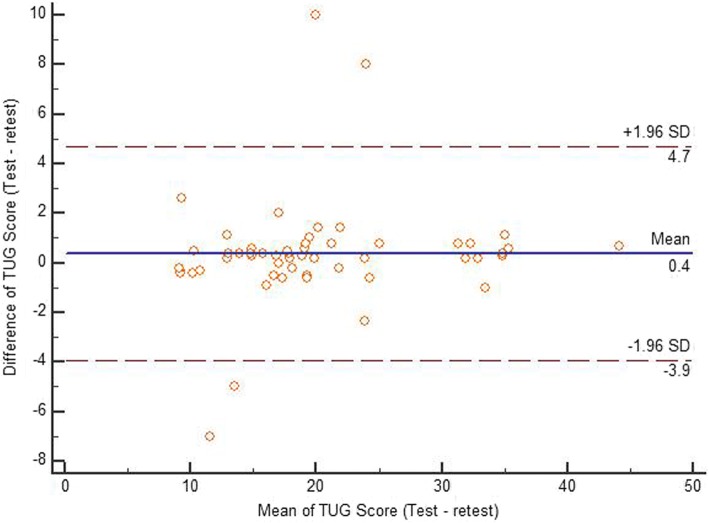
Bland-Altman plot showing reliability of the Timed up and go test (TUG)

**Fig. 2:**
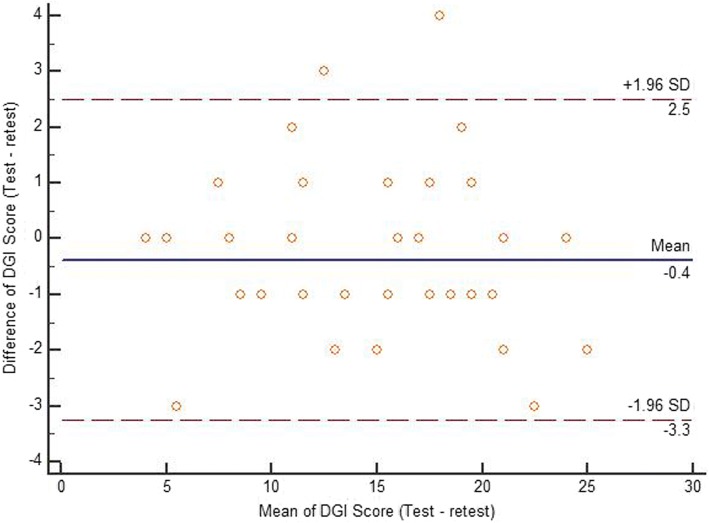
Bland-Altman plot showing reliability of the Dynamic gait index (DGI)

**Fig. 3:**
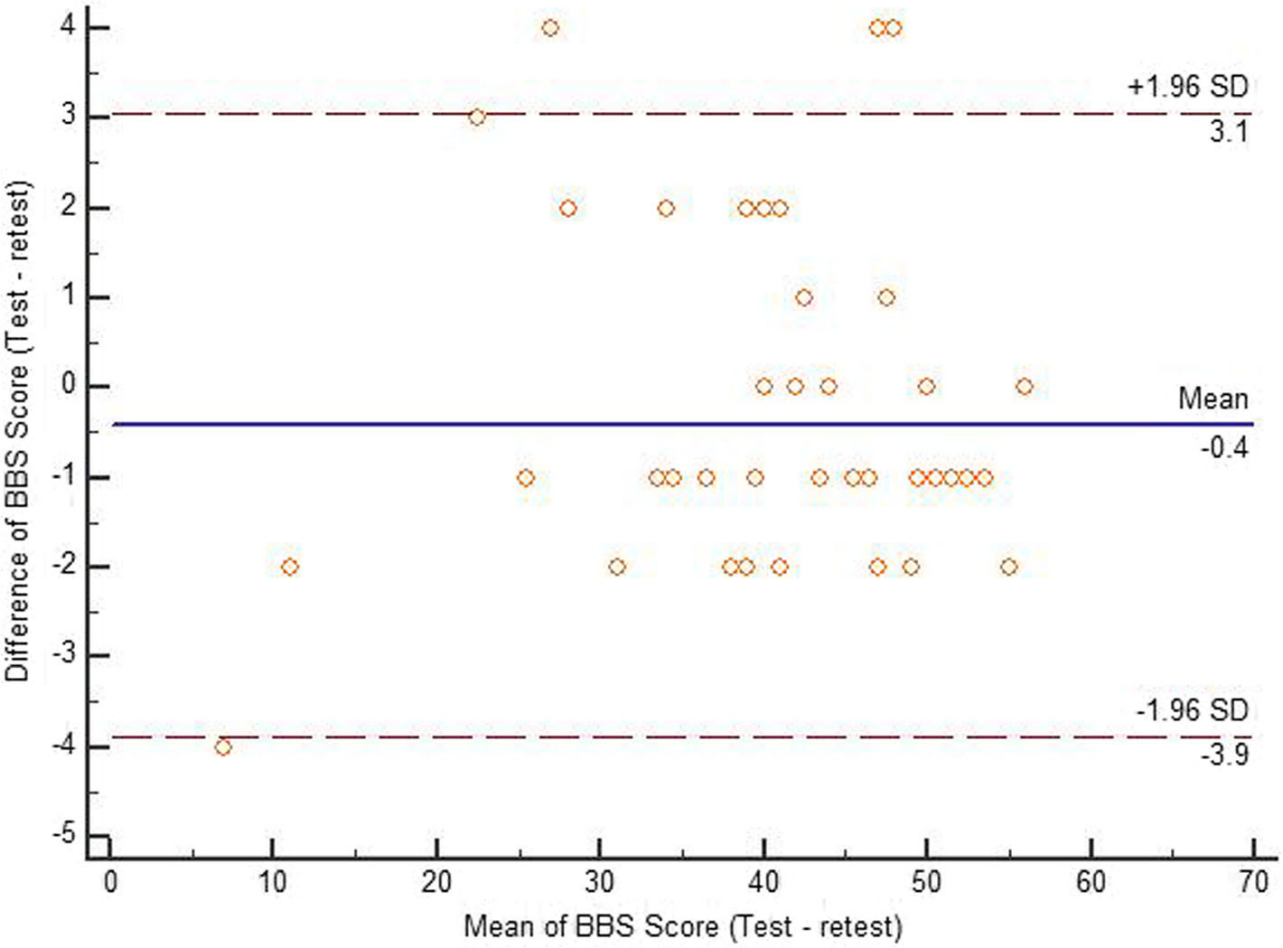
Bland-Altman plot showing reliability of the Berg balance scale (BBS)

Table 3 details the test-retest data. Test-retest reliability of the TUG, DGI, and BBS scores were found to be excellent. The Bland-Altman limit of agreement of each scale is presented in Figs. 1, 2 and 3 showing a reasonable agreement between test – retest score of each scale. The SEM of the TUG, DGI, and BBS were 1.16, 0.71, and 0.98, respectively. The MDC of the TUG, DGI, and BBS were 3.2, 1.9, and 2.7, respectively (as shown in Table 3). There was a significant correlation found between the DGI and BBS (first reading [r] = 0.75; second reading [r] = 0.77), TUG and BBS (first reading [r] = −.52; second reading [r] = −.53), and the TUG and DGI (first reading [r] = 0.45; second reading [r] = 0.48), respectively (Table 4). Table 5 details the correlations between demographic variables with the three scales. The participant’s age was significantly correlated with DGI and BBS scores. Duration since stroke was significantly correlated with DGI scores. Type of stroke was significantly correlated with BBS scores. The responsiveness data of the three scales are given in Table 6. The change in responsiveness of the TUG, DGI, and BBS was moderate from baseline to discharge.

**Table 3:** ICCs, Confidence Intervals, Standard error of measurement (SEM), and minimal detectable change (MDC) of the timed up and go test (TUG), the dynamic gait index (DGI), and the berg balance scale (BBS) (test-retest)

	TUG	DGI	BBS
ICC (95% CI)	0.98 (0.97–0.99)	0.98 (0.97–0.99)	0.99 (0.98–0.99)
SEM	1.16	0.71	0.98
MDC	3.2	1.9	2.7

**Table 4:** Correlation among the timed up and go test (TUG), dynamic gait index (DGI), and the berg balance scale (BBS) at different reading

	TUG vs DGI	DGI vs BBS	TUG vs BBS
First Reading	−0.45**	0.75**	−.52**
Second Reading	− 0.48**	0.77**	−.53**

**Correlation is significant at the 0.01 level;

**Table 5:** Correlations among demographic variables and the three scales

	Age	Height	Weight	Affected side	Duration since stroke	Type of stroke
TUG	0.225	− 0.017	0.161	−0.001	−.020	0.202
DGI	−0.386*	0.164	−0.093	0.020	−.337*	−0.166
BBS	−0.545**	0.213	0.093	0.112	−.045	−0.312*

*Correlation is significant at the 0.05 level; **Correlation is significant at the 0.01 level

*TUG* Timed up and go test, *DGI* Dynamic gait index, *BBS* Berg balance scale

**Tables 6:** Responsiveness of timed up and go test (TUG), the dynamic gait index (DGI), and the berg balance scale (BBS)

Indices of responsiveness	TUG	DGI	BBS
Baseline	20.1 ± 8.3	14.9 ± 5.3	41.4 ± 10.9
Discharge	16.9 ± 7.9	17.4 ± 4.8	45.7 ± 8.6
Mean difference	3.11	2.52	4.34
Pooled Standard Deviation	8.18	5.02	9.85
Standard Deviation of paired differences	5.87	2.82	5.37
*Effect size (ES) using pooled SD (95% CI)	0.38 (−0.57 – − 0.08)	0.50 (0.31–0.73)	0.44 (0.23–0.67)
Standardized response mean (SRM) (95%CI)	0.53 (−1.01 – − 0.03)	0.89 (0.47–1.30)	0.81 (0.47–1.01)

*Cohen’s d

## Discussion

Balance and mobility are the most important functional limitations in patients with chronic stroke [40]. A variety of balance and mobility related outcomes tools available, some of them designed to measure the multiple-task outcome, while others measure a single task. For a measure to be useful, it should be easy to administer, valid, reliable, and responsive [41, 42]. In the present study, the reliability, validity, and responsiveness of the TUG test, BBS, and DGI for measuring balance and mobility was assessed in patients with chronic stroke. The test-retest reliability of the three scales including, TUG test, DGI, and BBS were excellent. Similarly, a previous study reported an excellent reliability of the TUG test and the total BBS score in patients with chronic stroke [16]. The reliability of the TUG test, total DGI scores, and total BBS score in the current study were similar or near to those reported in previously published studies [7, 16, 43]. Jonsdottir and Cattaneo [7] reported an ICC value of 0.96 for total DGI scores. Hiengkaew et al. [16] reported an ICC value of 0.95 for the total BBS scores. In addition, Lin et al. [42] reported a similar test-retest reliability of the total DGI scores of individuals with chronic stroke. Blum and Korner-Bitensky [44] reported a slightly higher test-retest reliability (ICC = 0.98) of the BBS in patients with stroke. In contrast, another study reported a lower reliability of the total BBS score (ICC = 0.88) in patients with chronic stroke [45]. Similarly, Flansbjer et al. [18] reported lower test-retest reliability (ICC = 0.95) of TUG test in patients with chronic stroke. However, these studies had a higher sample size than the current study. In addition, the former study had a high proportion of left-sided hemiplegia in their participants compared to the current study in which right-sided hemiplegia was dominant. Furthermore, Ng and Hui-Chan [34] reported slightly lower test-retest reliability (ICC = 0.95) of the TUG test in patients with chronic stroke. However, Ng and Hui-Chan [34] study had a smaller sample size including only 11 subjects with chronic stroke.

In the present study, the SEM value of TUG test was slightly higher than the total DGI scores (1.16 vs. 0.71) and the total BBS scores (1.16 vs. .98). Similarly, a previous study reported lower SEM (0.97) for the total DGI scores in patients with chronic stroke [7]. Flansbjer et al. [45] reported a lower SEM score for the total BBS scores than those reported in the present study (1.49 vs. 1.93). However, Hiengkaew et al. [16] reported a higher SEM score for the TUG test than that in the present study (3.22 vs. 1.16). In the present study, the MDC value of the TUG test was lower than that in a previously published study (3.2 vs. 7.8) [16]. Similarly, the MDC value of the total BBS scores was lower than that in a previously published study (2.7 vs 4.7) [16].

In the present study, a good positive correlation was found between the DGI and BBS, and a moderate negative correlation was found between the TUG and BBS. Jonsdottir and Cattaneo [7] reported a moderate positive correlation between the DGI and BBS, and a moderate negative correlation between the DGI and TUG test. Although, in the present study, there was a slightly lower negative correlation found between the TUG test and DGI, this confirms the concurrent validity of these scales. In addition, Vistamehr et al. [46] reported a moderate positive correlation between the DGI and BBS total scores. In a future study of large cohort might give a better correlation among these scales.

The TUG, DGI, and BBS displayed a moderate degree of responsiveness from baseline to discharge, indicating they can adequately detect patients’ recovery following an intervention. However, DGI showed a better responsiveness compared to the TUG and BBS. A previous study reported an acceptable level of responsiveness of BBS at various stages of recovery in patients with stroke [47]. Another study reported a moderate level of responsiveness of DGI in detecting changes at the 5-month period of intervention in patients with chronic stroke [43]. No previous study had reported the responsiveness of the TUG test in detecting changes following an intervention in patients with chronic stroke. The current study indicates that the three scales are able to detect changes in patients with chronic stroke undergoing outpatient physiotherapy.

Generalization of the present results should be limited to the individuals with chronic stroke who could walk at least 10 m with or without a walking aid. Since it is not possible to score 4 points using a walking aid in DGI assessment, it becomes a 3-points scale for those participants who used such aids. This could results a better reliability of this scale. In addition, the degree of plantar flexor tone was not measured, which could affect the present results. Furthermore, lack of data about the premedical stroke history, exact stroke location and size may affect the scale interpretation. Since fewer female patients participated in this study, gender influence was not considered. However, this could have some impact on the overall responsiveness of each scale. It is recommended to examine the treatment effect on the DGI, TUG, BBS scores, muscle strength, the degree of spasticity, and gait parameters in prospective studies in patients with chronic stroke. Additionally, the small sample size limits the validity of the results. Therefore, future parametric studies are needed with larger sample size to confirm this finding and to compare these scales to one another.

## Conclusions

The test-retest reliability of the TUG, BBS, and DGI was excellent. The DGI demonstrated better responsiveness than TUG and BBS. The results of the present study support the use of these scales for measuring balance and mobility in patients with chronic stroke.
